# Pregnancy-related acute kidney injury leads to hypertension, reduced kidney function and cognitive impairment in postpartum rats

**DOI:** 10.3389/fphys.2024.1468793

**Published:** 2024-11-25

**Authors:** Ashley Griffin, Jamie Szczepanski, Shauna-Kay Spencer, Lucia Solis, Teylor Bowles, Reanna Robinson, Jan M. Williams, Patrick B. Kyle, Kedra Wallace

**Affiliations:** ^1^ Program in Neuroscience, University of Mississippi Medical Center, Jackson, MS, United States; ^2^ Department of Obstetrics and Gynecology, University of Mississippi Medical Center, Jackson, MS, United States; ^3^ Department of Pharmacology & Toxicology, University of Mississippi Medical Center, Jackson, MS, United States; ^4^ Department of Pathology, University of Mississippi Medical Center, Jackson, MS, United States

**Keywords:** AKI, CKD, cognition, HELLP syndrome, memory, postpartum, pregnancy, renal injury

## Abstract

**Introduction:**

Women with hypertensive disorders of pregnancy such as HELLP (hemolysis, elevated liver enzyme, low platelet) Syndrome are affected by acute kidney injury during pregnancy (PR-AKI) at higher rates than women without hypertension. Both hypertensive disorders of pregnancy and Acute Kidney Injury (AKI) outside the context of pregnancy have been associated with an increased risk of developing Chronic Kidney Disease (CKD) and cognitive impairment. In our current study, we set out to determine if PR-AKI led to the development of CKD and impaired cognition in the postpartum period and if HELLP syndrome exacerbates the impairments.

**Methods:**

Using timed-pregnant Sprague Dawley rats, on gestational day (GD) 12, mini-osmotic pumps infusing anti-angiogenic factors were surgically placed in the intraperitoneal cavity to induce HELLP. On GD18, AKI was induced via bilateral renal reperfusion ischemia surgery. Mean arterial pressure and birth outcomes were used to assess the global effects of AKI, and liver enzymes were used to assess HELLP. CKD was assessed by measuring glomerular filtration rate (GFR), urinary output, and renal fibrosis. Anxiety-like behaviors, object recognition memory, spatial memory, and avoidance memory were assessed via behavioral experiments.

**Results:**

HELLP + AKI rats demonstrated more evidence of renal injury, hypertension, and behavioral deficits compared to normal pregnant animals. In addition, AKI had a negative impact on birth outcomes and maternal survival.

**Conclusion:**

HELLP + AKI together led to evidence of persistent hypertension, progressive renal dysfunction, and cognitive impairment, which were exacerbated compared to AKI or HELLP alone. These findings suggest that PR-AKI in the presence of a hypertensive disorder of pregnancy, such as HELLP, leads to the development of CKD, cognitive dysfunction, and hypertension.

## 1 Introduction

Acute kidney injury during pregnancy (PR-AKI) is a serious obstetric complication that is characterized by diminished renal function and is associated with an increased risk of both maternal and fetal morbidity and mortality (Hall and Conti-Ramsden, 2019). Overall, the incidence of PR-AKI has been decreasing in developed countries due in part to improvements in prenatal care; however, the incidence of PR-AKI in the United States has been increasing over the past decade (Shah et al., 2020). The reasons for this increase are proposed to be in part due to increased rates of obesity, older maternal age at birth, and increasing rates of hypertensive disorders during pregnancy (Piccoli et al., 2018; Taber-Hight and Shah, 2020).

Hypertensive disorders of pregnancy, such as preeclampsia and HELLP (hemolysis, elevated liver enzymes, low platelets) syndrome, have been reported to be a major contributor to the development of acute kidney injury (AKI) in the antenatal and prenatal periods (Szczepanski et al., 2020a; Cooke et al., 2018; Choudhary et al., 2024). A study by Novotny et al. (2020) evaluated 1,276 women diagnosed with either HELLP syndrome or preeclampsia for the incidence of AKI and reported that 14.4% of HELLP patients developed AKI vs. 4.7% of preeclamptic women (Novotny et al., 2020). Similar results were reported following a meta-analysis indicating that women with HELLP syndrome are at a higher risk for developing AKI compared to women without HELLP syndrome (Liu et al., 2020). HELLP syndrome is a multi-systemic disease occurring in 0.2%–0.6% of women during pregnancy and in 10%–20% of women with preeclampsia (Wallace et al., 2018a; Campos et al., 2016).

It was previously believed that women with PR-AKI recovered renal function shortly after delivery; however, more recent studies report partial renal recovery to worsening renal function following PR-AKI (Ayansina et al., 2016; Berhe et al., 2024; Liu et al., 2017). Patients with renal impairment have reports of cognitive dysfunction and dementia (Kendrick et al., 2019; Theilen et al., 2016; Lipnicki et al., 2017). Women with a history of severe hypertensive disorders of pregnancy, such as preeclampsia and/or HELLP syndrome, are also at increased risk of developing impairments in cognitive function and/or dementia (Theilen et al., 2016; Arafa et al., 2024). Therefore, it stands to reason that the combination of a hypertensive condition and PR-AKI would increase the risk of cognitive dysfunction in the postpartum period. We have previously reported that renal ischemia-reperfusion during pregnancy leads to decreased renal function in both normal pregnant and HELLP rats (Szczepanski et al., 2020b). In the current study, we test the hypothesis that PR-AKI leads to the progression of chronic kidney disease (CKD) and cognitive decline in the postpartum period and that the combination of PR-AKI with HELLP syndrome exacerbates this decline.

## 2 Materials and methods

Studies utilized female timed-pregnant Sprague-Dawley rats (Charles River; Frederick, MD; 230–250 g upon arrival at gestational day (GD) 11). Our study exclusively examined female rats because the diseases modeled are only relevant in females. Rats were maintained on a standard rat chow diet (0.4% sodium) and housed in a temperature-controlled room with a 12:12 reverse light: dark cycle throughout the study. All experimental procedures were in accordance with the National Institutes of Health guidelines for use and care of animals and were approved by the Institutional Animal Care and Use Committee at the University of Mississippi Medical Center (protocol 2022–1198).

### 2.1 Experimental animal models

On GD12, a subset of rats, while under isoflurane anesthesia, had mini-osmotic pumps (model 2002, Alzet Scientific Corporation; Cupertino, CA) inserted intra-peritoneally to allow for the infusion of soluble fms-like tyrosine kinase 1 and soluble endoglin (4.7 and 7 μg/kg respectively, R&D Systems) to induce the HELLP syndrome phenotype as previously described (Wallace et al., 2014; Bean et al., 2016; Morris et al., 2016). AKI was induced on GD18 via a bilateral renal ischemia-reperfusion procedure for 45-min, as previously described (Szczepanski et al., 2020b) in a subset of normal pregnant rats (n = 16) and a subset of HELLP rats (n = 14) while they were under isoflurane anesthesia. Briefly, rats undergoing AKI were placed on an AIMS™ Thermo-controlled surgical platform before an abdominal midline incision was made. Once the incision was made, the uterine horns containing rat pups were gently pulled from the cavity until the kidneys were exposed. The renal pedicles were isolated and occluded with microvascular clamps for 45 min during which time the rat pups were placed within the abdominal cavity and the entire exposed abdominal area was covered with a warm saline soaked gauze pad, which was kept moist during the 45-min occlusion period. Ischemia was confirmed by a lack of pulsation in the renal pedicles and the fading of the kidneys from bright red to a dark purple–black color. Following the occlusion period, microvascular clamps were removed and the abdomen closed with suture. Normal pregnant (NP; uncomplicated pregnancy; n = 14) and HELLP (n = 14) rats not undergoing AKI received SHAM abdominal surgeries at this time. For rats in the SHAM group, they received an identical 45-min procedure except for renal pedicle occlusion. All rats were allowed to recover in cages with fresh and dry bedding that were placed half on half off a heating pad and under a heat lamp.

**FIGURE 1:**
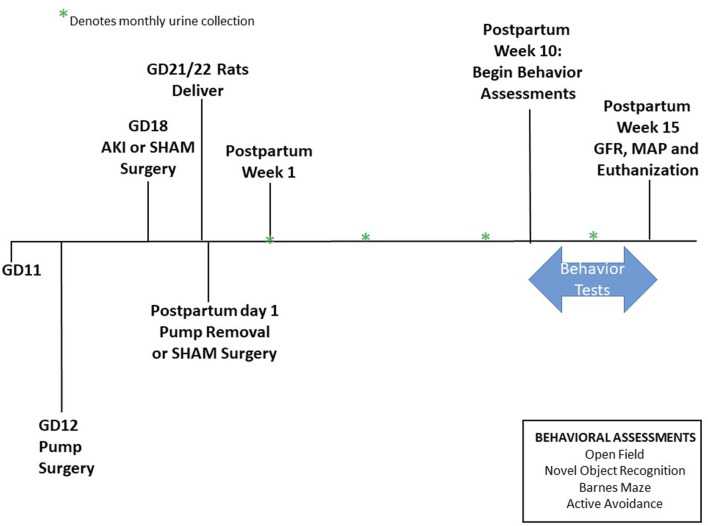
Study Timeline. Animals arrive on GD11. Pump surgery occurred on GD12, and AKI surgeries occurred on GD18. Pups were born between GD21-22. Pumps were removed on postpartum day 1. Urine collection began at postpartum week 1 then once a month following. Behavioral experiments were performed between postpartum week 10 until 15. GFR was measured at postpartum week 15 before euthanization.

Rats delivered between GD21-22 without intervention and within 24 h of pup delivery all rats underwent abdominal surgery to either remove the HELLP pump (to mimic the decrease in placental soluble fms-like tyrosine kinase 1 and soluble endoglin that occurs after delivery in human pregnancies complicated with HELLP syndrome) or to serve as a SHAM abdominal surgery (rats receive an abdominal midline incision and are then immediately closed with suture). At this time, all pups were also removed from the home cage. At the time of delivery, the number and weight of live and dead pups was recorded, and pup survival rate at birth was calculated as the percentage of live pups divided by total pups. Beginning at PPW1 (postpartum week; postpartum days 5–7), longitudinal information (urine output) was collected and then repeated monthly; Figure 1 outlines the experimental timeline.

### 2.2 Assessment of postpartum anxiety and cognition

Open Field. Animals had a one-hour acclimation period before behavioral testing, and all behavior was recorded using the Noldus Ethovision system unless noted. Anxiety-like behavior was assessed via the open field (40 cm × 50 cm × 50 cm), where rats were allowed to explore the center and edges of an arena for 20 min (Wallace et al., 2018b). The more time animals spend along the edges, the greater their anxiety.

Novel object recognition (NOR). Recognition memory was assessed via the NOR task as previously described (Wallace et al., 2022). Rats were placed in a chamber box with two identical objects for 5 min. The amount of time spent exploring the objects was recorded. After an inter-trial interval, rats were placed in the chamber and allowed 5 min to explore 1 familiar object and 1 novel object. Time spent initially interacting with 2 objects was recorded, followed by recording the time spent interacting with either the same object, a novel object, or no interactions. The recognition index was calculated ((time exploring novel object/time exploring both objects) × 100) to determine memory recall.

Barnes Maze. The Barnes Maze was used to evaluate spatial memory over the course of 5 days, as previously described (Wallace et al., 2022). Briefly, following a daily adaptation period in the goal box (i.e., escape chamber) and holding cage period, rats were placed directly in the center of the platform and allowed to explore the maze. Over 5 days, rats were allowed 300 s to find the escape chamber using three stationary spatial cues. The time needed to acquire the target hole was analyzed. If the escape chamber was not located, the subject was returned to the home cage at the end of the trial, and the time marked as 301 s.

Active Avoidance (AA). Rectangular shuttle boxes (Med Associates, St. Albans, VT), consisting of two identical compartments, were used for AA learning. After a 5-min acclimation period in the shuttle box, an audible tone and cue light were presented in the compartment of the shuttle box in which the animal was located. If, after 5 s, the animal had not moved to the other side of the shuttle box, an electrical shock through the grid floor occurred, lasting for 20 s or until the animal moved to the other shuttle box, whichever occurred first. All animals underwent fifty AA sessions on a single day, with an intertrial interval of 30 s, for a total of 9 days. Avoidance, escape, and response latency were assessed automatically through laser beam detection during every session. Each session included fifty trials.

### 2.3 Assessment of CKD

Following overnight urine collection, the total volume excreted and time period of collection were recorded to determine the urine output rate (mL/hour), and collected urine was frozen at −20°C. Proteinuria was assessed via the bicinchoninic acid assay (Thermo Fisher, Waltham, MA), creatinine and albuminuria were measured via commercially available kits following the manufacturer’s instructions (BioAssay Systems, Hayward, CA and Sigma Aldrich, St. Louis, MO respectfully). At PPW15, the glomerular filtration rate (GFR via clearance of circulatory FITC-sinistrin) was measured to assess renal function in a subset of rats (n = 5–8) as previously described (Szczepanski et al., 2020b). At the time of euthanization, PPW15, the left kidney (n = 5/rats per group) was fixed in 10% buffered formalin and stored in 70% ethanol prior for paraffin embedment. 4µM sections were stained with Masson’s trichrome, and the degree of renal fibrosis was assessed around the renal cortex. All images (10 per kidney section per rat) were digitally captured with a Nikon Eclipse 55i microscope equipped with a Nikon DS-F11 color camera (Nikon; Melville, NY) and analyzed using the NIS-Elements D3.0 software program to assess renal fibrosis (percentage of the image stained blue) (Szczepanski et al., 2020b).

### 2.4 Assessment of hypertension

All rats underwent carotid artery catheter insertion for mean arterial pressure (MAP) measurement while under anesthesia during PPW15, as previously described (Wallace et al., 2018b). The following day, MAP was continuously recorded for a 30-min period. After MAP was assessed, rats were anesthetized, and whole blood was collected to determine lactate dehydrogenase (LDH, hemolysis), aspartate transaminase (liver injury), and platelet counts and saved for future analysis. Maternal organs, such as the kidney, were stored in 10% buffered paraformaldehyde and submitted for paraffin fixation.

### 2.5 Statistical analysis

Two-way ANOVA with Tukey’s multiple comparisons *post hoc* analysis was used to assess the effect of AKI vs. HELLP. *P* < 0.05 was considered statistically significant. Mixed model analysis was used to assess the statistical relationship in longitudinal data. In the multiple comparison tests, group effect refers to NP vs. HELLP and treatment AKI vs. SHAM. Results are presented as ± standard error mean, and statistical analysis was conducted using GraphPad Prism 10.

## 3 Results

### 3.1 Maternal AKI decreases pup birthweight

All pups weighed significantly less relative to NP pups (Table 1). There was a significant AKI effect (*p* = 0.005) and AKI x group interaction (*p* = 0.05) on pup birthweight, with pups born to NP + AKI rats weighing the least compared to all other groups. The percent of pups born alive was calculated, and there was a significant AKI effect on pup survival (*p* = 0.03; Table 1); however, *post hoc* analysis did not indicate differences between specific groups. There were no statistically significant differences in litter size among the rats (*p* = 0.45).

**TABLE 1:** Pup birthweight was assessed at the time of birth among experimental groups.

	NP (n = 14)	HELLP (n = 14)	NP + AKI (n = 16)	HELLP + AKI (n = 14)
Pup Birthweight (g)	6.47 ± 0.09	6.15 ± 0.05^a^	5.15 ± 0.08^a,b,c^	5.9 ± 0.09^a^
Pup Survival (%)	92.4 ± 4	93.9 ± 3.8	76.2 ± 10.6^a,b^	77.5 ± 9.6^a,b^
Litter Size	10.15 ± 3.1	12.7 ± 2.7	8.5 ± 4.6	8.14 ± 4.8

^a^denotes *p* < 0.05 vs. NP; ^b^denotes *p* < 0.05 vs. HELLP; ^c^denotes *p* < 0.05 vs. HELLP + AKI.

### 3.2 AKI leads to increased anxiety and memory impairments following pregnancy

**FIGURE 2:**
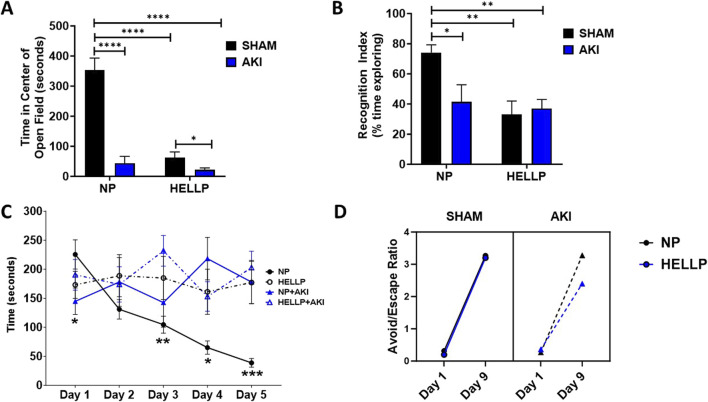
Behavioral Outcomes. HELLP and PR-AKI animals spent less time in the center compared to NP animals **(A)**. HELLP and PR-AKI animals had a decrease in recognition memory index compared to NP animals **(B)**. HELLP and PR-AKI animals spent more time to escape overall compared to NP animals exhibiting spatial memory deficits **(C)**. All groups showed improvement in the ratio to avoid/escape between day 1 and 9 **(D)**. * - ****p < 0.05–0.00001.

There was a significant AKI effect (*p* < 0.0001), group effect (*p* < 0.0001), and group x AKI interaction (*p* < 0.0001) on the time spent in the center of the open field. HELLP and AKI rats spent significantly less time in the open area vs. NP rats (*p* < 0.0001; Figure 2A), indicative of anxiety-like behavior in these rats. AKI further decreased the time spent in the open area in HELLP rats (*p* = 0.02). Memory was also tested with a battery of learning and memory tests. In the NOR test, there was a significant group effect (*p* = 0.006) and group x AKI effect (*p* = 0.03). In comparison to NP rats, there was a significant decrease in the recognition of familiar objects amongst HELLP (*p* = 0.006), NP + AKI (*p* = 0.03), and HELLP + AKI (*p* = 0.003; Figure 2B) rats. HELLP and AKI rats had significant impairments in spatial memory as assessed by the Barnes Maze (Figure 2C). There was a significant day x group (*p* = 0.0002) and AKI (*p* = 0.02) effect. On the initial day, PR-AKI animals escaped faster than NP animals (*p* = 0.03); however, beginning with day 2, NP rats escaped and were able to complete the task faster than other groups (Supplementary Table S1).

Learning and memory were tested in the AA test. There was a significant session effect (*p* < 0.0001, *p* < 0.0001) and treatment effect (*p* < 0.0001, *p* < 0.0001) in both the number of avoids and escapes rats completed during the testing period (Supplementary Figures S1B, D). There were no statistically significant differences between groups (*p* = 0.45) or in response to AKI (*p* = 0.09) in latency to avoid the shock in AA learning (Supplementary Figure S1A). Results were similar with latency to escape the shock as there was not a statistical difference between groups (*p* = 0.34) or in response to AKI (*p* = 0.33; Supplementary Figure S1C). There was a positive correlation between the number of sessions completed and latency to avoid among NP (r^2^ = 0.55, *p* = 0.02) and NP + AKI rats (r^2^ = 0.60, *p* = 0.01). To further evaluate the relationships, we compared the ratio of avoids/escapes from the beginning of the trial (day 1) to the end of the trial (day 9). There was a significant difference in the ratio between days (*p* < 0.0001), with all groups improving in the number of avoidances relative to escapes by the final session (Figure 2D).

### 3.3 Renal injury following PR-AKI persists into the postpartum period

**FIGURE 3:**
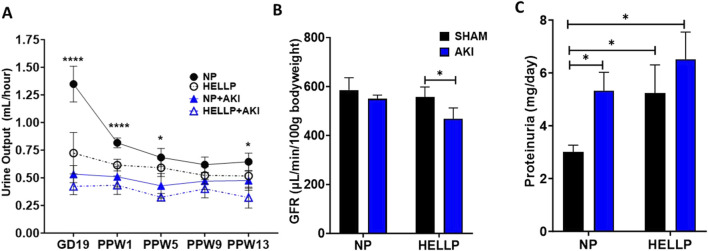
Renal Function Measurements. Urine output was assessed via metabolic cages throughout the study and was decreased in response to HELLP + AKI from GD19 until PPW5 **(A)**. GFR was significantly decreased in HELLP + AKI vs. HELLP animals at PPW15 **(B)**. Proteinuria was significantly increased in all groups relative to NP rats at PPW13 **(C)**. AKI = Acute Kidney Injury, GFR = Glomerular Filtration Rate, HELLP = Hemolysis, elevated liver enzyme, low platelets Syndrome, NP = Normal Pregnant *-**** denotes *p* < 0.05–0.0001.

**FIGURE 4:**
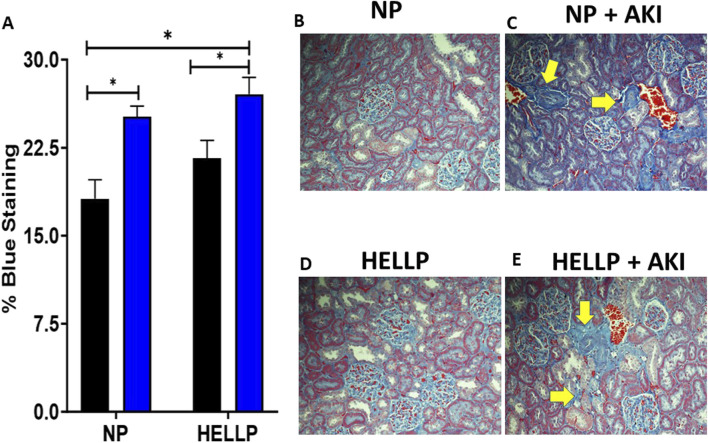
Renal Fibrosis is increased following AKI. Renal fibrosis was significantly increased in AKI vs. non-AKI animals **(A)**. Representative renal trichrome images for NP **(B)**, NP + AKI **(C)**, HELLP **(D)** and HELLP + AKI **(E)** are shown with the yellow areas indicating areas of increased fibrosis. * denotes *p* < 0.05.

Renal function was assessed by GFR to determine if AKI during pregnancy was associated with chronic renal dysfunction. There was a significant time (*p* = 0.002), AKI (*p* < 0.0001), group (*p* = 0.0007), and time x AKI interaction (*p* = 0.03) on urine output. Throughout the study, urine output remained decreased in all groups except NP rats with HELLP + AKI rats being the most affected (Figure 3A). Relative to NP rats at GD19 (*p* < 0.0001) and PPW1 (*p* = 0.0009), all groups had significantly decreased urine output, however by PPW5, only AKI rats remained significant (*p* = 0.01), with only HELLP + AKI rats still having a significant decrease in urine output by the end of the study (*p* = 0.02). When GFR was assessed, there was a significant AKI effect (*p* = 0.02) but no group effect (*p* = 0.28). Post-hoc analysis indicated that HELLP + AKI rats had significantly reduced renal function vs. HELLP rats (*p* = 0.04; Figure 3B). There was a significant group effect (*p* = 0.05) on urine creatinine that was collected at the end of the study. Post-hoc analysis indicated that HELLP + AKI rats excreted significantly more creatinine relative to NP rats (*p* = 0.001; Supplementary Table S2). There was a significant group effect (*p* = 0.007) and treatment effect (*p* = 0.02) in albuminuria levels. Post-hoc analysis indicated that HP (*p* = 0.01) and HELLP + AKI rats (*p* = 0.007) excreted significantly more albumin relative to NP rats (Supplementary Table S2). Proteinuria was also measured and was found to be significantly increased in HELLP, NP + AKI, and HELLP + AKI rats relative to NP rats (Figure 3C). Renal fibrosis, a common pathological finding in CKD, was assessed in kidney sections collected from euthanized rats. There was a significant AKI effect (*p* = 0.002; Figures 4B–E) in the amount of fibrosis. Both NP + AKI (*p* = 0.04) and HELLP + AKI (*p* = 0.02; Figure 4A) have significantly more fibrosis relative to NP rats. HELLP + AKI rats also had significantly more fibrosis vs. HELLP rats (*p* = 0.04).

### 3.4 Hypertension and thrombocytopenia persist into the postpartum period following PR-AKI

MAP was evaluated to determine if PR-AKI worsened hypertension in the postpartum period. There was a significant group effect on MAP (*p* = 0.01) in which both HELLP (*p* = 0.02) and HELLP + AKI (*p* = 0.0002) rats had significantly higher MAP relative to NP rats (Table 2). Hemolysis was elevated with a significant group effect (*p* = 0.02), with HELLP rats having significantly elevated LDH relative to NP (*p* = 0.03), NP + AKI (*p* = 0.02), and HELLLP + AKI (*p* = 0.04; Table 2) rats. There was not a significant AKI effect (*p* = 0.55), group effect (*p* = 0.63), or AKI x group interaction (*p* = 0.92) on levels of liver enzymes. There was a significant AKI effect (*p* = 0.03), group effect (*p* = 0.005), and AKI x group interaction (*p* = 0.002) in platelet count. HELLP (*p* = 0.0006) and AKI rats (*p* = 0.003, *p* = 0.003) had significantly fewer platelets compared to NP rats (Table 2). Survival was also assessed as AKI rats died unexpectedly throughout the study period (Supplementary Figure S2). By the end of the study, four (28.9%) HELLP, six (37.5%) NP + AKI, and four (28.9%) HELLP + AKI rats had unexpected deaths (*p* = 0.09), whereas there were no deaths among NP rats.

**TABLE 2:** Mean arterial pressure (MAP) and parameters of HELLP syndrome assessment.

	NP (n = 11)	HELLP (n = 10)	NP + AKI (n = 9)	HELLP + AKI (n = 9)
MAP (mmHg)	114 ± 7.5	124.67 ± 9.3^a^	122.42 ± 16.94	131.44 ± 7.6^c^
LDH (IU/mL)	659.13 ± 149.47	1,431.13 ± 263.14^a^	654 ± 92.62^d^	753.27 ± 170.21^d^
AST (IU/mL)	122.56 ± 18.51	112.4 ± 8.63	131.44 ± 24.35	124.67 ± 15.73
Platelets (µL)	9.4 × 10^5^ ± 1.3 × 10^5^	2.7 × 10^5^ ± 1.1 × 10^5c^	3.4 × 10^5^ ± 9.2 × 10^4b^	3.9 × 10^5^ ± 8.3 × 10^4b^

^a–c^ Denotes *p* < 0.05–0.005 relative to NP, rats; ^d^ denotes *p* < 0.05 vs. HELLP, rats; LDH, lactate dehydrogenase; AST, aspartate aminotransferase.

## 4 Discussion

Existing studies on renal function and cognitive impairment following hypertensive pregnancies complicated with renal disease are limited and mainly focus on the impact on offspring. However, as the prevalence of hypertensive disorders of pregnancy and PR-AKI is increasing, it is important to determine the course of impairment and renal function in women affected by the combination of AKI in the setting of a hypertensive disorder of pregnancy (Shah et al., 2020; Leonard et al., 2024). In the current study, we used animal models of PR-AKI and HELLP + AKI and reported that rats experiencing AKI have evidence of worsening renal function, hypertension, and cognitive impairment following pregnancy.

This is the first study to examine the impact of AKI and a severe hypertensive disorder of pregnancy on the association between postpartum renal function and cognition. We and others have observed cognitive impairment following hypertensive disorders of pregnancy in rodent models (Wallace et al., 2022; Johnson et al., 2022). Here, we observed that PR-AKI was associated with increased anxiety and impairments in memory as rats spent less time in the open area of the open field, spent less time interacting with a novel object, and were not able to significantly locate the escape box in the Barnes maze. Rats with a history of HELLP syndrome had similar findings of increased anxiety and memory impairments; however, when AKI was induced in the setting of HELLP, there was a further increase in anxiety and in learning as evidenced by the decreased ratio of avoids/escapes in the AA test. These findings are consistent with previous reports demonstrating anxiety, reduced learning, and memory in rodents following AKI or CKD (Silva et al., 2024) or HELLP syndrome (Wallace et al., 2022). In a rat model of CKD, animals showed deficits in the performance of hippocampal-dependent memory tasks (Yu et al., 2022). Clinically, decreased renal function is associated with cognitive impairment and an increased risk of developing dementia (Elias et al., 2009; Tsai et al., 2017). The results from the current study suggest that PR-AKI impairs cognition and increases anxiety and that hypertensive disorder of pregnancy worsens the response to AKI.

Preeclampsia is associated with an increased risk of developing end-stage renal disease (Vikse et al., 2008; Khashan et al., 2019), and women who develop HELLP syndrome are even more prone to need dialysis and remain hypertensive in the postpartum period (Sibai and Ramadan, 1993; Gul et al., 2004). In the current study, we demonstrated that at 15 weeks postpartum, renal fibrosis remained significantly elevated in rats who had undergone PR-AKI compared to normal pregnant rats, indicating that renal damage persisted. In addition to intrinsic renal damage, we demonstrated that renal function remained impaired in rats who had undergone PR-AKI, as evidenced by oliguria. Furthermore, the presence of experimental HELLP prior to AKI increased the degree of renal injury in CKD. The extent of renal damage in HELLP rats was not as severe as what was seen in HELLP + AKI rats. These findings are similar to what has been reported among women with HELLP not complicated with AKI as Ye et al. reported decreased creatinine among HELLP patients compared to HELLP + AKI patients (Ye et al., 2019). Clinical studies examining the long-term effects of HELLP syndrome are sparse, however a study by Habli et al. reported that among HELLP patients without AKI, only 2.4% had evidence of renal injury following pregnancy (Habli et al., 2009). These studies, combined with the current data, suggest that in the absence of AKI, HELLP alone may not be enough to warrant progression to severe renal injury in the short postpartum period. However, more studies are needed to fully understand the long-term complications of renal injury following pregnancies complicated with HELLP syndrome.

The link between hypertension, stroke, and other cardiovascular diseases among patients with CKD is well-established (Matsushita et al., 2010). The same is true for women with a history of hypertensive pregnancies (Amaral et al., 2015); however, while evidence indicates that hypertensive disorders of pregnancy are a major contributor to the incidence of PR-AKI (Szczepanski et al., 2020a), there is little data on long-term effects of PR-AKI on blood pressure. A recent study by Muteke et al. (2023) evaluating postpartum patients in Uganda reported resolution of hypertension or proteinuria among preeclamptic women with and without AKI at 7 days postpartum (Muteke et al., 2023); however, another study reported renal injury and proteinuria during pregnancy were associated with both hypertension and persistent proteinuria in women with severe preeclampsia (Murali et al., 2024). One study reports that at 6 months postpartum, Dahl rats with a previous history of superimposed preeclampsia had evidence of renal injury but were no longer hypertensive (Turbeville et al., 2019). While renal function was not examined, we previously reported that within the first 3 postpartum months, rats with a history of preeclampsia were not hypertensive, whereas HELLP rats were hypertensive (Wallace et al., 2022). Collectively, these results indicate different cardiovascular effects due to different hypertensive disorders of pregnancy.

We also examined the biochemical parameters of HELLP syndrome to determine if AKI worsened these conditions and found that AKI contributed to thrombocytopenia. Decreased platelet count is frequently reported among patients with CKD and is believed to contribute to some of the disease morbidity (Jain et al., 2021). Interestingly, there was a significant decrease in LDH levels in AKI rats relative to HELLP rats. This is in contrast to what was previously reported when LDH was measured during pregnancy (24 h following AKI surgery) where NP + AKI and HELLP + AKI rats had significantly increased LDH (Szczepanski et al., 2020b). In fact, the majority of clinical studies have found a positive correlation between renal disease symptomology and elevated serum LDH (Xiao et al., 2023; Zhang and Shi, 2021). In the transplant literature, patients with lower LDH levels were more likely to have an immune rejection to kidneys vs. patients with high LDH who had evidence of ischemic damage (Sandor et al., 2024). Along these same lines, mice have also been reported to have different responses to the isoforms of LDH and after chronic renal injury will produce less LDH relative to mice without renal injury (Osis et al., 2021). We have not yet explored the oxidative stress and immune pathways that are well-defined in renal injury patients and models, nor have we looked at the function and isoforms of LDH but these are all mechanisms that could explain why PR-AKI leads to a decreased LDH during the postpartum period in the current study.

When AKI or hypertensive disorders complicate pregnancies, there is a higher rate of stillbirth/perinatal death and lower birthweight among affected offspring (Liu et al., 2017; Wang et al., 2024). We investigated birth outcomes following PR-AKI and observed that AKI led to decreased birthweight compared to NP controls, and while both the percent of alive pups at birth and total litter size were reduced in PR-AKI rats vs. NP rats, it did not reach statistical significance. PR-AKI also increased maternal mortality compared to both HELLP and NP rats, which is similar to what is seen clinically (Shah et al., 2020; Ye et al., 2019).

This study is limited by the lack of investigation into possible mechanisms that may underlie the increased progression of renal dysfunction, anxiety, and cognitive changes. Increased permeability of the blood-brain barrier in AKI and HELLP syndrome has been proposed to occur due to several different mechanisms, such as inflammation, oxidative stress, and increased levels of uremic toxins (Wallace et al., 2018a; Lu et al., 2015). For instance, the uremic toxin indoxyl sulfate accumulates following long-term renal injury and has been reported to increase blood-brain barrier permeability in animals with CKD (Bobot et al., 2020), which is negatively associated with cognitive function in patients with CKD (Lin et al., 2019). Our group has recently published data demonstrating that the administration of indoxyl sulfate during pregnancy increases the permeability of the blood-brain barrier of pregnant rats, a phenomenon similar to what occurs in pregnant rats with HELLP syndrome (Bean et al., 2016; Griffin et al., 2023). Indoxyl sulfate has also been implicated in contributing to anxiety in both animal and clinical studies (Karbowska et al., 2020; Brydges et al., 2021). Additionally, the inflammatory pathway has been implicated to contribute to both the progression of HELLP syndrome and renal injury. We have previously published data demonstrating that during pregnancy anti-angiogenic factors sFlt-1 and sEng are increased in NP + AKI and HELLP + AKI rats, as are renal CD3^+^CD4^+^ T cells (Szczepanski et al., 2020b). This suggests that PR-AKI contributes to alterations in the immune pathway that could have negative consequences on the blood-brain barrier and long-term changes in behavior (Ijomone et al., 2021; Varatharaj and Galea, 2017). Future studies will focus on the mechanisms that contribute to the current findings.

## 5 Conclusion

In summary, the results of the current study, taken together with those from other studies, indicate the need for further investigations into the long-term effects of PR-AKI with and without a pre-existing hypertensive condition. We demonstrated that anxiety and cognitive impairment was accompanied by decreased renal function in rats with a history of PR-AKI, which was exacerbated in HELLP rats. Importantly, these models of PR-AKI open the doors for future mechanistic studies to better understand the pathophysiological mechanism that leads to the progression of renal impairment and memory changes in the postpartum period.

## Data Availability

The raw data supporting the conclusions of this article will be made available by the authors, without undue reservation.

## Glossary

AA: active avoidance
AKI: acute kidney injury
CKD: chronic kidney disease
GD: gestational day
GFR: glomerular filtration rate
HELLP: hemolysis, elevated liver, low platelet
LDH: lactate dehydrogenase
MAP: mean arterial pressure
NOR: novel object recognition
NP: normal pregnant
PPW: postpartum week
PR-AKI: pregnancy-related acute kidney injury
