# Supplementary material for: Pregnancy-related acute kidney injury leads to hypertension, reduced kidney function and cognitive impairment in postpartum rats
Source: Front Physiol. 2024 Nov 25;15:1468793. doi: 10.3389/fphys.2024.1468793 (PMC11625802; doi:10.3389/fphys.2024.1468793)
Supplement: Supplementary file 1 [file Image1.pdf]

Supplementary Material

1 Supplementary Data

Supplemental Table 1. Time taken to complete Barnes Maze is listed in seconds.

Day	NP	HELLP	NP+AKI	HELLP+AKI
1	225.5±83.8[d]	173.18±69.74	144.80±67.921	190.56±102.45
2	130.99±55.55	188.79±108.71	178.15±125.87	173.87±117.81
3	104.62±48.62	185.03±111.50	142.54±127.65	231.73±102.89[b]
4	65.07±37.78	161.21±116.38[a]	218.29±109.64[b]	152.99±98.38 [a]
5	38.76±24.79	177.23±109.36[a]	177.93±111.24[b]	202.91±108.41[c]

[a-c]denotes $p<0.05$-0.0005 vs. NP respectively; [d]denotes $p<0.05$ vs. NP+AKI.

Supplemental Table 2. Urine creatinine and albumin values measured at PPW13, the last metabolic cage time point prior to euthanization. Data represented as mean±standard error mean.

Analyte	NP	HELLP	NP+AKI	HELLP+AKI
**Creatinine (mg/dL/hr)**	261.49±57.06	414.84±93.13	491.3±120.68	667.8±96.7[b]
**Albuminuria (g/dL/hr)**	4.86±0.58	7.57±0.6 [a]	7.1±0.8	9.93±1.66 [b]

[a,b] denotes $p<0.05$ and $p<0.005$ vs. NP

2 Supplementary Figures and Tables

Supplemental Figure 1.

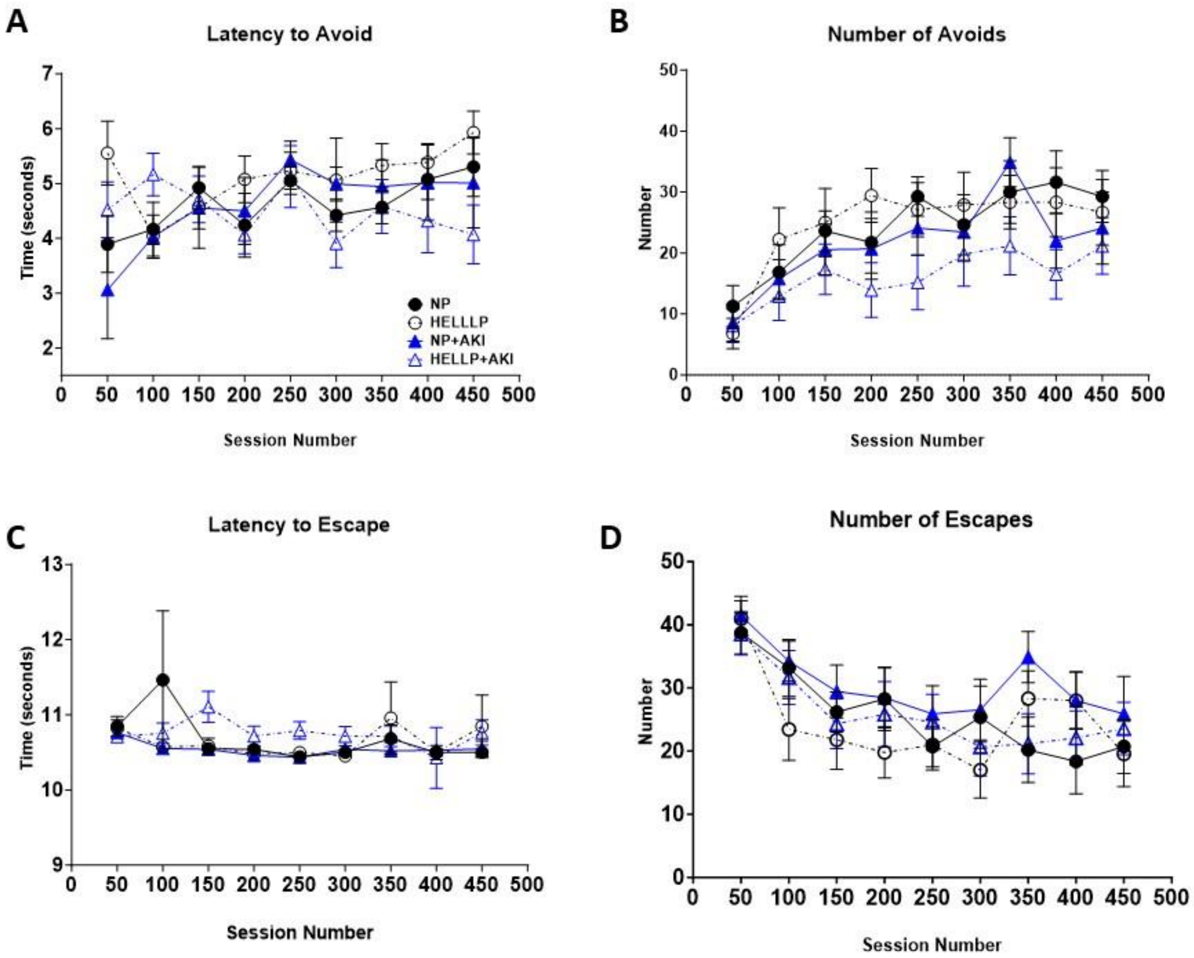

Supplemental Figure 1. Active avoidance. (A) Latency to Avoid, (B) Number of Avoids, (C) Latency to Escape (D) Number of Escapes.

Supplemental Figure 2.

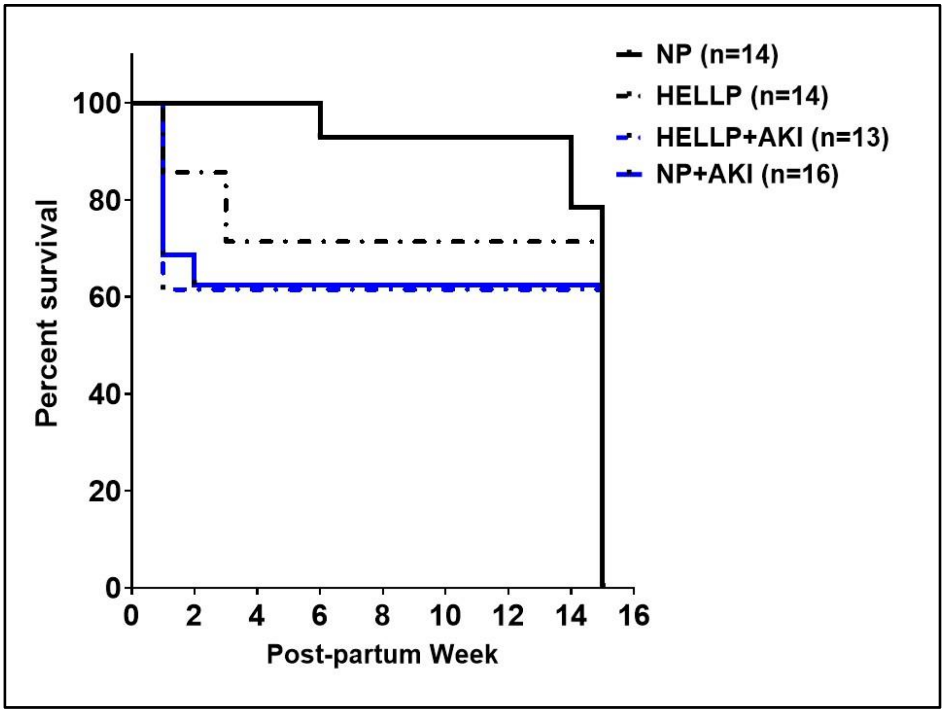

Supplemental Figure 2. Survival Curve. Survival was assessed throughout the study.
